# A dataset of non-pharmaceutical interventions on SARS-CoV-2 in Europe

**DOI:** 10.1038/s41597-022-01175-y

**Published:** 2022-04-01

**Authors:** George Altman, Janvi Ahuja, Joshua Teperowski Monrad, Gurpreet Dhaliwal, Charlie Rogers-Smith, Gavin Leech, Benedict Snodin, Jonas B. Sandbrink, Lukas Finnveden, Alexander John Norman, Sebastian B. Oehm, Julia Fabienne Sandkühler, Jan Kulveit, Seth Flaxman, Yarin Gal, Swapnil Mishra, Samir Bhatt, Mrinank Sharma, Sören Mindermann, Jan Markus Brauner

**Affiliations:** 1 Manchester University NHS Foundation Trust, Manchester, UK; 2 Future of Humanity Institute, University of Oxford, Oxford, UK; 3 Medical Sciences Division, University of Oxford, Oxford, UK; 4 Faculty of Public Health and Policy, London School of Hygiene and Tropical Medicine, London, UK; 5 Department of Health Policy, London School of Economics and Political Science, London, UK; 6 The Francis Crick Institute, London, UK; 7 School of Life Sciences, University of Warwick, Coventry, UK; 8 OATML Group (work done while at OATML as an external collaborator), Department of Computer Science, University of Oxford, Oxford, UK; 9 Department of Computer Science, University of Bristol, Bristol, UK; 10 Mathematical, Physical and Life Sciences (MPLS) Doctoral Training Centre, University of Oxford, Oxford, UK; 11 Medical Research Council Laboratory of Molecular Biology, Cambridge, UK; 12 University of Cambridge, Cambridge, UK; 13 University of Essen, Essen, Germany; 14 Department of Mathematics, Imperial College London, London, UK; 15 Oxford Applied and Theoretical Machine Learning (OATML) Group, Department of Computer Science, University of Oxford, Oxford, UK; 16 Medical Research Council (MRC) Centre for Global Infectious Disease Analysis, School of Public Health, Imperial College London, London, UK; 17 Abdul Latif Jameel Institute for Disease and Emergency Analytics (J-IDEA), School of Public Health, Imperial College London, London, UK; 18 Section of Epidemiology, Department of Public Health, University of Copenhagen, Copenhagen, Denmark; 19 Department of Statistics, University of Oxford, Oxford, UK; 20 Department of Engineering Science, University of Oxford, Oxford, UK

**Keywords:** Epidemiology, Viral infection

## Abstract

During the second half of 2020, many European governments responded to the resurging transmission of SARS-CoV-2 with wide-ranging non-pharmaceutical interventions (NPIs). These efforts were often highly targeted at the regional level and included fine-grained NPIs. This paper describes a new dataset designed for the accurate recording of NPIs in Europe’s second wave to allow precise modelling of NPI effectiveness. The dataset includes interventions from 114 regions in 7 European countries during the period from the 1st August 2020 to the 9th January 2021. The paper includes NPI definitions tailored to the second wave following an exploratory data collection. Each entry has been extensively validated by semi-independent double entry, comparison with existing datasets, and, when necessary, discussion with local epidemiologists. The dataset has considerable potential for use in disentangling the effectiveness of NPIs and comparing the impact of interventions across different phases of the pandemic.

## Abstract (summary)

**Table:** 

Measurement(s)	Government non-pharmaceutical interventions against Covid-19
Technology Type(s)	Interpretation by researchers
Sample Characteristic - Organism	Homo sapiens

## Background & Summary

During the first half of 2020, countries responded to SARS-CoV-2 transmission with a range of non-pharmaceutical interventions (NPIs) that were generally implemented at a national level and have been analysed extensively using an array of public datasets^1,2^. In response to a resurging wave of SARS-CoV-2 transmission during the second half of 2020, many European countries implemented NPIs at a local level instead. Some countries used a tier system to implement groups of measures in local areas. While researchers have produced multiple NPI datasets^3–10^, accurate modelling of the pandemic’s second wave has been limited by the use of NPI definitions and data from the first wave. During the second and subsequent waves, governments introduced bans on gatherings with small group sizes (below 10), closures of specific sections of the economy (retail, night clubs, gastronomy), and curfews. In addition, these interventions were often targeted at smaller geographical areas in response to incidence rates. It is crucial for the ongoing management of NPIs that policymakers have effectiveness estimates based on more recent and accurate data. High-quality data requires thorough validation with expert input and interpretation of often ambiguous NPIs. To address these challenges, we collected a novel dataset to allow for analysis of interventions during the second SARS-CoV-2 wave in Europe from August 2020 to January 2021^11^.

The dataset presented here has several features that make it ideal for high-quality modelling and reuse. The precise categorisation of NPIs allows for greater differentiation than previous datasets in terms of restrictions on gathering size, gathering type, and types of business closures. More precise definitions of NPIs are instrumental for disentangling the most effective NPIs which can prove critical for policymakers. Additionally, the regional granularity of the dataset enables analysis of a period when interventions were often implemented at the subnational level. The manual collection of data and semi-independent manual validation ensures higher quality than can be obtained with purely automated data collection.

**Fig. 1:**
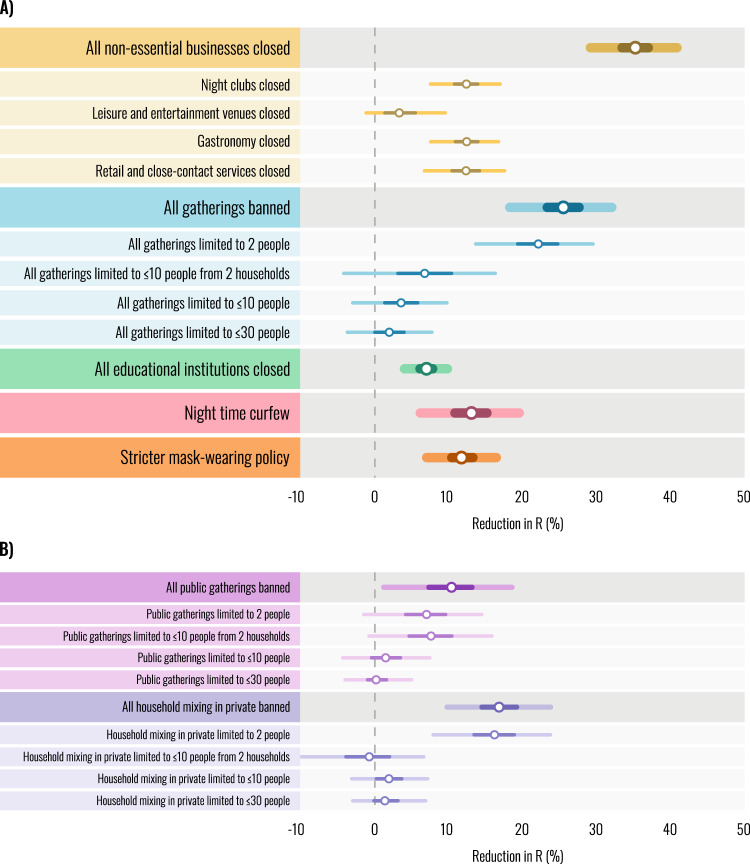
The effectiveness of interventions in Europe’s second wave as estimated by Sharma *et al*.^11^. Percentage reductions in the reproduction number R shown, this is the instantaneous reproduction number Rt. Rt is the expected number of secondary infections arising from a primary infection at time t. Markers indicate posterior median estimates, lines indicate the 50% and 95% posterior credible intervals. A negative 1% reduction refers to a 1% increase in R (**A**) Effectiveness of the main interventions included in Sharma *et al*.^11^. Intervention names preceded by “All” show the combined effect of multiple interventions. For example, “All gatherings banned” shows the combined effect of banning all public gatherings and all household mixing in private. (**B**) Individual effectiveness estimates for gathering types, separated into public gatherings and household mixing in private. Figure reproduced from Sharma *et al*.^11^.

This dataset has multiple valuable applications. As many countries continue to face high incidence of SARS-CoV-2 and policymakers weigh different options for reducing transmission, this dataset enables researchers to disentangle the effectiveness of individual interventions. For example, using the dataset in combination with local case and mortality data, Sharma *et al*. use a hierarchical Bayesian transmission model to estimate the effectiveness of 17 NPIs and identify the closure of specific businesses and the strictest restrictions on gatherings as the most effective at reducing transmission (see Fig. 1)^11^. Moreover, as it includes NPIs during the second wave, the dataset can be used to estimate differences in the effectiveness of NPIs over time. For example, Sharma *et al*. find that, relative to the first wave, the combined effect of all NPIs appears smaller during the second half of 2020 than during the first wave of epidemics in Europe^2,11^. Understanding the changing effectiveness over time is not only crucial for addressing continuing SARS-CoV-2 outbreaks but also for the management of future emerging epidemics. In addition to enabling the comparison of different interventions, this dataset has already been used to adjust for NPIs when analysing the effect of seasonality and mask use on SARS-CoV-2 transmission^12,13^. The regional data may also be used to investigate the effect of NPIs on social, health, and economic outcomes in neighbouring regions. Finally, the custom data collection methods described in this paper can be used both to expand the regional and temporal scope of the dataset as well as to inform data collection for other datasets.

Researchers may consider using this dataset for several reasons. One reason is if they require subnational data for this period. Another reason is if they require additional context in the form of comments, quotes, and original sources for every entry. Furthermore, if researchers are investigating the changing response to SARS-CoV-2 over time they may benefit from our NPI definitions which are tailored to the second wave, and supply our dataset with more granular differentiation in types of gatherings and business closures. Finally, this dataset will be best suited for researchers who require extensively validated data as it is the only dataset that is fully validated with semi-independent double entry.

## Methods

These methods are expanded versions of descriptions in our related work by Sharma *et al*.^11^.

### Dataset design process

Every data collection process begins with the decision about what data to collect. In the following, we describe how we addressed key decisions and judgement calls in our dataset design. This process may serve as inspiration or partial guideline for future data collection efforts.

First, we conducted an open-ended, exploratory data collection to identify (i) the main NPIs implemented in Europe’s second wave, (ii) suitable NPI definitions that faithfully represent these interventions while enabling consistent coding across countries, and (iii) the level of administrative division–such as districts, administrative regions, or states–on which NPIs were implemented in each country. To this end, we first defined a set of broad NPI categories and collected all changes of any NPI in these categories, as well as any other major NPIs, in several candidate countries. We collected NPI data from various levels of administrative divisions to identify the largest level of division for which NPI implementation no longer varied within a region. The exploratory collection demonstrated that several NPI definitions commonly used in first-wave analyses were too coarse for the second wave. For example, the strictest level of gathering bans recorded in other datasets is commonly a limit of 10 people or fewer^6,14^. However, many of the important NPIs in Europe’s second wave happened *within* that category—limits of 6, 4, or 2 people, or a ban on all gatherings—so that more fine-grained categories were needed. More granular categories were similarly needed for other NPIs, such as mask-wearing policies and business closures. Beyond contributing to finding suitable NPI definitions, further benefits from the exploratory data collection included (i) information on the practical feasibility of the data collection (e.g. whether it was prohibitively difficult to find reliable data on particular NPIs), (ii) scoping of unforeseen challenges, and (iii) the construction of a set of best-practice guidelines for the main data collection.

Based on the results from the exploratory data collection, we finalised the dataset design with input from various team members and external experts. The rationale was that design decisions, such as which NPIs/countries/regions to include and exclude, require making complex tradeoffs and thus benefit from multiple perspectives and group-decision-making. First, we aggregated the results of the exploratory collection and created a written proposal on all design decisions. Then, team members gave written feedback on the proposal, and key design decisions were discussed with external experts (researchers with experience in NPI data collection and local epidemiologists). Finally, we finalised design decisions in a conference call with consensus from multiple team members.

## Dataset Summary

We collected chronological data on NPIs that were in place in 7 European countries. The dataset contains over 5,500 entries in 114 regions of analysis. Each entry contains the NPI start date and end date. In addition to one or more sources (government websites and universities, legal documents, and/or media reports) we also include quotes and comments providing context. Further details on dataset characteristics are available in Table 1.

**Table 1:** Dataset characteristics.

Country	Number of Regions	Administrative Divisions
Austria	9 (whole country)	States
Czech Republic	14 (whole country)	Administrative regions
England	15 (stratified random sample)	NUTS 3 statistical regions
Germany	15 (stratified random sample)	Districts
Italy	21 (whole country)	Administrative regions
Netherlands	25 (whole country)	Safety regions
Switzerland	15 (stratified random sample)	Cantons

Showing the number of administrative divisions per region. The period of analysis was from 1 August 2020 to 9 January 2021.

### Data window

The data window starts on 1 August 2020, to cover some of the period before the NPIs of the second wave started to enable before/after comparisons, and to cover the reopening of schools and universities after the summer holidays. The data window ends on 9 January 2021.

### Country selection

We included European countries for which data on daily reported cases and deaths (necessary for NPI intervention effectiveness modelling) were publically available at the same geographical resolution at which the country implemented NPIs (Austria, the Czech Republic, England, Germany, Italy, the Netherlands, Switzerland). Other countries were excluded because granular case and/or death data was not publicly available (e.g. Denmark, Portugal) or because NPIs were implemented on an extremely granular level in the country, making data collection infeasible (e.g. Spain with roughly 8000 municipalities).

### Region selection

During the exploratory data collection we identified the appropriate level of geographical granularity for data collection. We used the regions of analysis that corresponded to the largest administrative division for which NPI implementations were identical within each region. This is nearly always correct, with only a few exceptions for mostly short periods such as within the Essex: Haven Gateway region when Braintree moved to Tier 3 and Colchester & Tendring remained in Tier 2. Where regions were subdivided in terms of restrictions we added further detail in the comments. The chosen administrative divisions are summarised in Table 1.

For Austria, the Czech Republic, Italy, and the Netherlands, we collected data from the whole country (9, 14, 21, and 25 regions of analysis). For England, Germany, and Switzerland we took a stratified random sample of 15 regions of analysis, as it was not feasible to collect data from all regions. Each of the 16 German states had different regulations for its districts. To reduce the work required for data collection, we sampled the 15 districts only from the four largest states (Northrhine-Westphalia, Bavaria, Baden-Württemberg, Lower Saxony). These four states make up 60% of the population.

A random sample ensures the geographic diversity of sampled regions within each country and reduces bias in the region selection that would limit the generalisability of modelling findings. To ensure sufficient diversity of included regions, the sample was stratified by each regions’ cumulative number of COVID-deaths in the first wave. Regions with relatively few cases provide less evidence about the underlying reproduction number (and thus NPI effects) and thus provide little useful signal for modelling work. Additionally, regions with few cases may be more strongly affected by importations from neighbouring regions. To address both these issues, we only included regions with at least 2000 reported cases during the analysis period.

### NPIs

NPIs were identified based on the process discussed above. We focused on clearly defined, major interventions that were implemented in many countries. The included NPIs, as well as their corresponding definitions, are listed in the data records section. We separately recorded restrictions on public and private gatherings, as well as indoor and outdoor gatherings, to cover the breadth of interventions used in Europe’s second wave.

We recorded different educational institutions separately and recorded holidays as closures in addition to closures due to SARS-CoV-2. When a school moved most of its teaching online, this was also counted as an institutional closure, however, we handled this differently for universities. For example, if a school moved most of its teaching online, we reasoned that from that point forward there would be minimal transmission between that school’s pupils, so we categorised this as school closure. This is supported by Eames *et al*. who identified a significant reduction in daily recorded encounters when children were on holiday, although contact with older adults and children from other schools increased^15^. However, the situation would be different in universities: A university might have moved most teaching online (as was the case in almost all universities), but students generally returned to their university town at the commencement of the academic year in September/October 2020, which would mean continued transmission in the area through students socialising and living together. For universities, we thus recorded both vacation times (in which most university students will not be residing in the university town) and periods when universities send students away from university towns (e.g. by closing university accommodation) as closures.

We recorded NPIs corresponding to closures of various types of businesses. As with other NPIs, we only recorded closures and not safety measures (such as restrictions on the number of customers that may enter at any time, shorter opening hours, restrictions on the sale of alcohol). We also excluded restrictions on hotels/lodging, as these have little influence on local transmission and therefore limited use for modelling the local impact of interventions. Finally, we recorded mandatory mask-wearing policies of several levels of strictness, as taken from the Oxford COVID-19 Government Response Tracker (OXCGRT)^6^, and evening/night-time curfews.

**Fig. 2:**
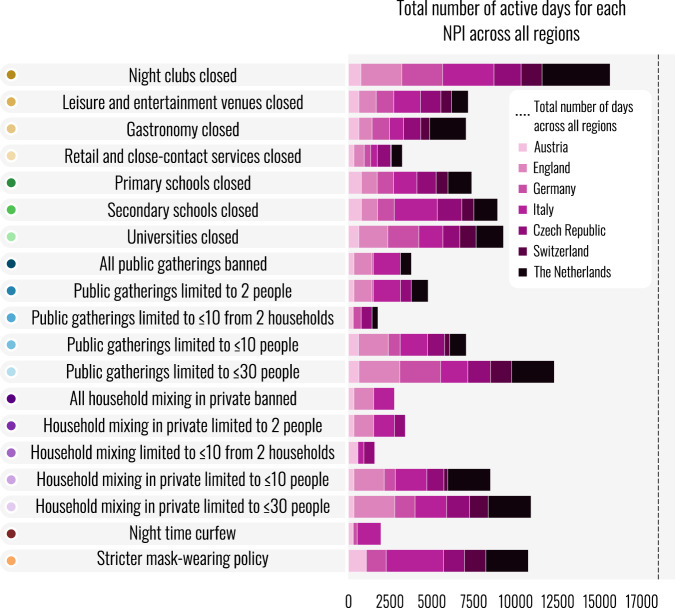
Overview of implementation of NPIs. The total number of days that each intervention was implemented, aggregated across regions but separated by country. The dashed vertical line indicates the total-number of region-days recorded in our dataset. Note that gathering and household mixing thresholds shown in this figure represent those used by Sharma *et al*.^11^, the dataset itself contains the precise limit on the number of attendants and households for each type of gathering. Figure reproduced from Sharma *et al*.^11^.

Several initially plausible NPIs were excluded after the exploratory data collection and further deliberation. We initially planned to record stay-at-home orders, as they had played a major role in Europe’s first wave. However, in the second wave of the pandemic, stay-at-home orders commonly had a multitude of exceptions, which covered most reasons for leaving the house. Often, these stay-at-home orders appeared exclusively in legislatory documents but were barely mentioned by media reports (e.g. in Bavaria, Germany), making it unlikely that residents would even be aware of them. Additionally, we considered including an NPI for shielding vulnerable populations but found it challenging to find definitions for this NPI that would be consistent across countries. An overview of the NPIs chosen, and their implementation across the selected countries is illustrated in Fig. 2.

## Data Collection

**Fig. 3:**
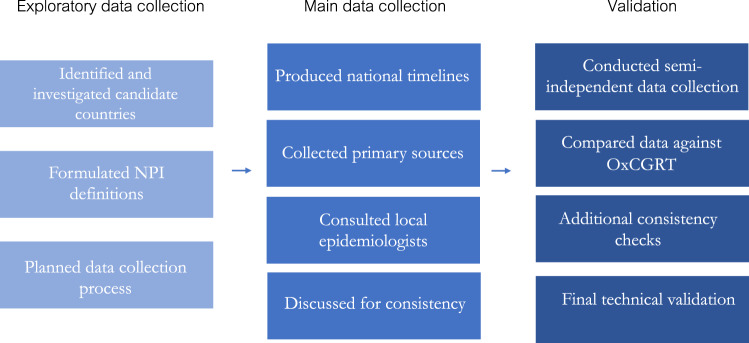
Process of data collection; from exploration to validation.

Nine researchers completed the manual data collection, using a set of data collection instructions including detailed NPI descriptions, suggested methods of collection, and heuristics for ambiguous cases. For each country, one or two researchers produced a national timeline. In most cases, two researchers then completed the initial manual data collection for NPIs in a single country(see Fig. 3). The first manual data collection involved adding dates, sources, and quotes for any change in the recorded NPIs during the period from the 1st of August 2020 - 9th of January 2021.

During this initial data collection, ambiguous cases and the general interpretation of NPIs were discussed on an online group working platform to ensure consistency. Throughout the data collection, local epidemiologists were consulted on the plausibility of the NPI timelines and provided additional context on the implementation of NPIs. The data collection took a total of 950 hours; 185 hours were required for the national timelines, 470 hours for collection of regional data, and a further 290 hours for validation. For further information on validation, see *Technical Validation*.

## Data Sources

The researchers used a range of primary sources during the data collection. Some NPI implementation dates were identified through internet searches and national COVID-19 response Wikipedia pages, although specific non-encyclopedic sources were always consulted to confirm each date. Local and national government press releases, legislation, and ordinances were used to confirm the meaning of specific interventions. Multiple sources were collected where available, in particular, the combination of government sources and media sources was used to confirm that proposed mandatory NPIs were implemented as planned. Media sources were also used in some cases to confirm the population were aware of any NPIs introduced.

Several countries including the UK, Austria, and Italy implemented a tiered approach to NPIs, with interventions of varying severity in each tier. For countries using such an approach, we investigated which components of the tier system met our NPI definitions. We then consulted local government and media sources to investigate if interventions were implemented as described by the tier system.

We used in-house language skills with team members fluent in English, German, Dutch, French and Danish to provide a correct interpretation of sources in the original language. Additionally, translation software was used for some media and government sources.

## Guiding heuristics for data collection

The researchers found ambiguities and unique circumstances in some areas of data collection. A key advantage of semi-independent double entry is that it provides a rigorous method of reducing individual bias in the interpretation of NPIs. To ensure consistency, a shared document was produced and shared with all data collectors. Key guiding heuristics of the data collection are summarised here, see also Supplementary Note 5 in Sharma *et al*.^11^.

### Record NPIs

Record NPIs when they affect most or all of the institutions, events, or people in question.

#### Gatherings

Restrictions on gatherings often included exceptions of certain types of gatherings (e.g. funerals or weddings) or areas (parks in England). We counted an NPI for gatherings as active if “most or all” of the types of gatherings or areas were included. In difficult cases, we consulted local epidemiologists and residents to determine the types of gatherings that were included in a particular NPI and whether this would account for “most or all” gatherings.

#### Educational institutions

Educational institutions could be closed, closed for some groups of students, open with the option for children to stay at home, or completely open. We determined that an NPI was in effect if “most or all” (>50%) of students in a particular group (primary, secondary, or university) were not attending the educational institution.

#### Sub-area NPI implementation

Occasionally, NPIs were introduced at a level of granularity smaller than the regions we collected. Therefore, a region may have multiple different NPIs active at the same time in local areas within the region. We recorded an NPI as active if most (>50%) of people in the region were affected by the NPI.

### Record interventions

Record interventions as they are expected to affect behavior.

#### Non-mandatory recommendations

Many countries had governmental recommendations that were not mandatory. For example, governments may recommend mask use in all indoor spaces but only legally require masks on public transport. In these cases, researchers followed a general rule of only including mandatory NPIs, because it is very hard to define what exactly counts or doesn’t count as a recommendation, and adherence to recommendations will vary greatly by country. Several exceptions to this rule were included in the cases of private gatherings in Italy, Austria, and the Northrhine-Westphalia state in Germany. In these cases, the regions did not have the legislative capacity to regulate private gatherings in a legally binding manner; however, discussions with local epidemiologists and residents were used in combination with media reports to confirm that these recommendations were followed by a significant majority of the population, and they were therefore included as active interventions in the dataset. The context surrounding these decisions was added to the comments.

#### The timing of implementations

We recorded an NPI as active from the date it is expected to have affected behaviour. If an NPI was announced as in place immediately we used the starting day of the announcement, even if legislation supporting the NPI was only passed as law later. In cases where the announcement for an NPI was made in the evening, we counted the NPI as active the next day, except in the case of nightclubs. For schools, we considered weekends as school closures, so that if a government announced that schools would close on Monday, the starting date for the NPI would be the preceding Saturday.

## Data Records

All data collected from this study has been published in a figshare repository^16^. The data is presented as it was collected, with a separate CSV file for each country. Alongside the primary, human-readable, CSV, we have also published a machine-readable CSV for ease of future use, which includes daily COVID cases and deaths, taken from various government sources https://github.com/MrinankSharma/COVID19NPISecondWave/blob/main/data/raw_data_w_sources/sources.md. This version includes a row for each day from 1st August 2020 to the 6th of January 2021, with columns for each of the NPIs. As it provides a row for each day in the time period, there are 154 rows for each of the 144 total regions collected, thus amassing 19,000 rows of data.

### Location

**Country:** Country of focus

**Region:** Region of analysis

### Limits on gatherings sizes and household mixing

**Limit on number of people (Public Outdoors):** The maximum number of individuals legally allowed to attend any gathering in an outdoor space that the general public has access to (such as a public park).

**Limit on number of households (Public Outdoors):** The maximum number of different households allowed to mix at any gathering in an outdoor space that the general public has access to (such as a public park).

**Limit on number of people (Public Indoors):** The maximum number of individuals legally allowed to attend any gathering in an indoor space that the general public has access to (such as a shopping mall).

**Limit on number of households (Public Indoors):** The maximum number of different households legally allowed to mix at any gathering in an indoor space that the general public has access to (such as a shopping mall).

**Limit on number of people (Private Outdoors):** The maximum number of individuals legally allowed to attend any gathering in a private outdoor space (such as a private garden).

**Limit on number of households (Private Outdoors):** The maximum number of different households legally allowed to mix at any gathering at a private outdoor space (such as a private garden)

**Limit on number of people (Private Indoors):** The maximum number of individuals legally allowed to attend any gathering in a private indoor space (such as a private house).

**Limit on number of households (Private Indoors)**: The maximum number of different households legally allowed to mix at any gathering at a private indoor space (such as a private house)

### Closures of face-to-face businesses

**Night clubs closed:** Night clubs closed

**Most or all gastronomy is closed or limited to take-away:** Businesses such as restaurants, bars, pubs, cafes are closed or limited to take-away.

**A large fraction of leisure and entertainment venues are closed:** Leisure venues, such as theatres, gyms, concert halls, casinos, indoor skating rinks, bowling alleys, are closed.

**Most or all retail shops and close-contact services are closed:** Most or all retail shops and close-contact services (such as hairdressers and massage parlours) are closed.

**All non-essential face-to-face businesses are closed:** All non-essential face-to-face businesses, as defined by each individual country, are closed.

**Curfew:** Most individuals must stay indoors during certain times of the day (typically at night). Exemptions for limited reasons usually exist.

### Schools

**Most or all primary schools (roughly 5 or 6 to 10 or 11 years) have stopped most or all in-person teaching:** Most or all primary schools (roughly 5 or 6 to 10 or 11 years) have stopped most or all in-person teaching.

**Most or all secondary schools (roughly 10 or 11 years to 17 or 18 years) have stopped most or all in-person teaching:** Most or all secondary schools (roughly 10 or 11 years to 17 or 18 years) have stopped most or all in-person teaching.

### Universities

**Most or all higher education institutions are on (summer) term-break, (Christmas) vacation, or have sent students away from the university town (e.g., by closing university accommodation)**. Most or all higher education institutions are on term-break, vacation, or have sent students away from the university town.

**How many universities does this local area have? 0, 1, or > 1?:** Number of universities in the region of analysis.

**How many students do the universities in this local area roughly have (combined)?**: Number of students attending universities in the local area (based on enrollment numbers).

**How many inhabitants does the local area roughly have?:** Number of inhabitants in the region of analysis.

### Mask-wearing

**Level of NPI (0–4):** OXCGRT scale of mask rule stringency applied here. These levels are:

- 1: Recommended mask-wearing
- 2: Required in some specified shared/public spaces outside the home with other people present, or some situations when social distancing is not possible
- 3: Required in most or all shared/public spaces outside the home with other people present or all situations when social distancing is not possible
- 4: Required outside the home at all times regardless

### Used for multiple columns

**Description of NPI:** Additional description of NPI, if applicable

**Start date:** Date NPI was first implemented

**Quotes and comments:** Quotes from sources showing the implementation and/or the relevant date. Comments explaining edge cases and any relevant information.

**Sources for start date:** Source for the start date

**End date:** Date NPI was first lifted

**Quotes and comments (if applicable)**: Quotes from sources showing the lifting of the regulation, implementation of a new regulation, and/or the relevant date. Comments explaining edge cases and any relevant information.

**Sources for end date (if applicable):** Source for the end date

**What is the reason they currently don’t teach: COVID, holidays, other?**: Identifying the reason for schools not being open

## Technical Validation

To ensure data quality, extensive validation tasks were carried out. Firstly, we compared our initial data collection against the national COVID-19 Government response tracker (OxCGRT)^6^. When additional sources were available (Italy and England), the data were validated against additional external sources^17,18^.

The NPI categories that corresponded to our collection were C1, C4, C6, and H6. If a data collector identified a difference between our data and the OxCGRT, they resolved this by revisiting both primary sources and if necessary discussing the difference with other members of the team or local epidemiologists. Finally, a researcher added the reasoning to the comments section.

Secondly, we completed a semi-independent double entry: For each country, we produced a copy of the spreadsheet that only included the sources, quotes, and comments. A researcher who had not taken part in the initial data entry for that country or local region then entered the dates on the new spreadsheet, without being able to see the dates that had initially been collected but while having access to the sources that other researchers had gathered for each region. The two spreadsheets were then compared and differences automatically flagged. The researchers who completed both entries then discussed, revisited primary sources, and resolved any differences.

Following this, a researcher manually compared a sample of data from all countries to ensure consistency in the application of NPI definitions and common judgement calls across countries. Finally, wherever possible, automated consistency checks were performed. E.g., we verified that all entered dates fall into the data collection period or that the chronological order between NPIs is consistent (start dates before end dates; earlier rows before later rows), that any very short gaps in NPI implementation are real and not data entry artefacts, and so on (see Fig. 3).

## Usage Notes

### Advantages of different NPI datasets

Researchers have produced several NPI datasets^3–9^ with different methods and goals and it is crucial for researchers to utilise data that best fits their research requirements. We recommend the use of alternative datasets if they require a larger selection of countries or countries with greater geographical and income variety, or if they seek to study the entire period of the SARS-CoV-2 pandemic^6–9^. Other datasets would also be more suitable for investigating economic measures which we do not include^5,19^.

## Data Availability

No code was used to generate the data.
